# Phenotypic PIA-Dependent Biofilm Production by Clinical Non-Typeable *Staphylococcus aureus* Is Not Associated with the Intensity of Inflammation in Mammary Gland: A Pilot Study Using Mouse Mastitis Model

**DOI:** 10.3390/ani11113047

**Published:** 2021-10-25

**Authors:** Jully Gogoi-Tiwari, Dorji Dorji, Harish Kumar Tiwari, Gayatri Shirolkar, Joshua W. Aleri, Trilochan Mukkur

**Affiliations:** 1 Curtin Health Innovation Research Institute, Curtin Medical School, Curtin University, Bentley, Perth, WA 6102, Australia; dorji1@graduate.curtin.edu.au (D.D.); gayatri.shirolkar@curtin.edu.au (G.S.); tk_mukkur@hotmail.com (T.M.); 2 School of Veterinary Medicine, College of Science, Health, Engineering and Education, Murdoch University, 90 South Street, Murdoch, WA 6150, Australia; J.Aleri@murdoch.edu.au; 3 Department of Microbiology, Jigme Dorji Wangchuck National Referral Hospital, Khesar Gyalpo University of Medical Sciences, Thimphu 11001, Bhutan; 4 Asia-Pacific Consortium of Veterinary Epidemiology, Sydney School of Veterinary Science, The University of Sydney, Camden, NSW 2570, Australia; harish.tiwari@sydney.edu.au; 5 Centre for Animal Production and Health, Future Foods Institute, Murdoch University, 90 South Street, Murdoch, WA 6150, Australia

**Keywords:** *Staphylococcus aureus*, non-typeable, biofilm, tissue damage, inflammation, bovine mastitis, mouse model

## Abstract

**Simple Summary:**

*Staphylococcus aureus*-associated human clinical infections are predominantly caused by the encapsulated strains, with non-typeable strains representing less than 25%. In contrast, 80% of the *S. aureus* from bovine mastitis cases are non-typeable as they do not possess the Capsular Types 1, 2, 5, and 8. In our previous studies, it was demonstrated that the extent of mammary tissue damage was associated with the strength of biofilms formed by encapsulated *S. aureus* strains. This study assesses the impact of biofilm formation, as a virulence factor of non-typeable *Staphylococcus aureus*, causing mammary tissue damage in a mouse mastitis model. The study demonstrates no association between the strength of biofilm production by non-typeable *S. aureus* and the mammary tissue damage. However, the mice infected with strong biofilm producing non-typeable *S. aureus* died 6h earlier than those infected with weak biofilm producing non-typeable *S. aureus* suggesting the role of biofilm in the advancement of the time of mice mortality.

**Abstract:**

Non-typeable (NT) *Staphylococcus aureus* strains are associated with chronic bovine mastitis. This study investigates the impact of biofilm formation by clinical NT *S. aureus* on cytokine production and mammary tissue damage by using a mouse mastitis model. Mice infected with two different NT *S. aureus* strains with strong and weak biofilm forming potential demonstrated identical clinical symptoms (moderate), minimal inflammatory infiltrates, and tissue damage (level 1 histopathological changes) in the mammary glands. However, the *S. aureus* load in the mammary glands of mice and the level of pro-inflammatory cytokines (IL-1β, IL-6, IL-12, IL-17 and IFN-γ) in serum were significantly higher (*p* ≤ 0.05) in those infected with the strong biofilm forming NT *S. aureus* strain. The level of IL-6 in sera samples of these mice was extremely high (15,479.9 ± 532 Pg/mL). Furthermore, these mice died in 24h of post infection compared to 30 h in the weak biofilm forming NT *S. aureus* infected group. The study demonstrates no association between the strength of PIA (polysaccharide intercellular adhesion)-dependent biofilm production by clinical NT *S. aureus* and mammary gland pathology in a mouse mastitis model. However, the role of biofilm in the virulence of *S. aureus* advancing the time of mortality in mice warrants further investigation.

## 1. Introduction

Presence of capsular polysaccharide (CP) and the biofilm forming ability of *S. aureus* are the major virulence determinants of the pathogen [1,2]. Capsular polysaccharide helps the organism to evade phagocytosis allowing the pathogen to persist in tissues and blood stream of the infected host [3]. The bacteria can invade and persist in both nonprofessional and professional phagocytic cells making the infection persistent through one lactation to the next [4,5,6,7]. The predominant capsular types in *S. aureus* are CP5 and CP8 and the prevalence of non-typeable (NT) *S. aureus* in human clinical isolates are less than 25% [3]. In contrast, *S. aureus* isolates from bovine mastitis cases are mostly non-typeable up to 86% [8], only 14–40% of the strains producing CP5 or CP8 [4,5,6,7]. While investigating the prevalence of *S. aureus* CP types associated with bovine mastitis cases in Australia and India, around 30% and 40% of the isolates were detected to be non-typeable, respectively [9]. NT *S. aureus* strains are associated with chronicity of infection as they can invade the mammary epithelial cells in higher numbers than the encapsulated ones leading to persistence of intramammary infection for longer duration [8].

Biofilm forming ability of *S. aureus* is an important virulent factor associated with bovine mastitis [10,11]. After entering the mammary gland, *S. aureus* adheres into the mammary epithelial lining and start forming biofilm [12]. Biofilm helps the pathogen to resist phagocytosis and antimicrobial agents by aggregation of colonies and formation of exopolysaccharide matrix leading to persistent infections of the mammary gland [13] and antibiotic treatment failure [14,15,16]. Bacteria in biofilm are 10–1000 times more resistant to antimicrobial agents than its planktonic form [17]. In addition, there is evidence of stimulation of biofilm formation by certain antibiotics such as tetracycline and erythromycin if used in sub inhibitory concentrations [18,19,20,21,22,23]. In addition, sub-inhibitory concentration of antibiotics is one of the major reasons of mastitis treatment failure leading to relapses and reinfections of mastitis in dairy cows [24]. The development of biofilm occurs in three steps including initial adherence, subsequent maturation and final detachment or dispersal and these steps are physiologically different from one another. The wide variety of extracellular virulence antigens of *S. aureus* have been reported to be associated with biofilm formation including PNAG (poly-N-acetylglucosamine), surface associated MSCRAMMS (microbial surface components recognizing adhesive matrix molecules) such as FnBPA, FnBPB, clfA, clfB, cna, Bap, ProteinA, SasG, phenol soluble modulins and BBP, extracellular DNA and toxins (hla and hlb) [18,19,20,21,22,23]. However, the widely described method of development of biofilm is the PIA (polysaccharide intercellular adhesion)/PNAG production which is synthesized by *icaADBC* operon of *S. aureus* [25,26]. *S. aureus* in biofilm is entirely a different entity compared to its planktonic form regarding phenotypic characteristics [27], antibiotic resistance pattern [12] and development of innate immune response [28]. While studying the immunogenicity of *S. aureus* in biofilm versus planktonic cultures in an experimental mouse mastitis model, we demonstrated that *S. aureus* in biofilm induced stronger and differential immune responses from its planktonic counterpart [29]. In a pilot study using a non-invasive mouse mastitis model, we noted severe mammary tissue damage with significantly higher levels of TNF-α in mice infected with strong biofilm producing CP8 positive *S. aureus* compared to weak biofilm former CP8 positive *S. aureus* [30].

Considering the predominance of NT *S. aureus* strains in bovine mastitis etiology, the ability of *S. aureus* to produce biofilm in the mammary gland and to induce distinct immune responses than its planktonic form, it is important to understand the extent of mammary tissue damage associated with intramammary infection with strong versus weak biofilm forming NT *S. aureus*. Few earlier studies focused on the virulence potential of *S. aureus* as slime versus non-slime producers [31], coagulase positive versus negative strains [32], and small colony variants of *S. aureus* [33]. However, there is paucity of knowledge about the impact of strength of in vitro biofilm formation by clinical NT *S. aureus* on the mammary gland pathology. To the best of our knowledge, no studies have been conducted to investigate the association between strength of biofilm formation by NT *S. aureus* and the mammary tissue damage due to production of inflammatory cytokines, the central mediators of inflammatory response during mastitis [34]. This study was undertaken to investigate the impact of biofilm formation by clinical NT *S. aureus* on cytokine production and mammary tissue damage. In our previous studies, it was discovered that damage caused to the mammary gland by infection with encapsulated *S. aureus* was associated with the strength of biofilm formation with an increase in TNF-α level [30]. The current study hypothesised that mice infected with NT *S. aureus* with strong biofilm forming potential will develop severe mammary tissue damage compared to the NT *S. aureus* with weak biofilm forming potential. The aim of this experiment, therefore, was to investigate association between PIA-dependent biofilm production by clinical NT *S. aureus* and mammary gland pathology by using a mouse mastitis model. 

The use of large animal models including cow, goat, and sheep, to study bovine mastitis has its associated problems such as cost and management, even using minimal number of animals. Besides, only limited number of hypotheses can be studied in large animals [35]. Mouse is still considered a suitable animal model for bovine mastitis research due to cost effectiveness, minimum management and similarity between mouse and cow’s mammary glands in respect to neutrophil infiltration and tissue damage [35]. Both the species have two pairs of anatomically and functionally independent mammary glands in inguinal region [36]. In addition to these two pairs, mouse has three additional pairs of mammary glands in the thoracic region which can be used to study additional parameters in mastitis research [36].

## 2. Materials and Methods

### 2.1. S. aureus Phenotypes

Two NT *S. aureus* strains isolated from mastitis cases of cows, with strong or weak biofilm forming potential, were used in this investigation (Table 1). Both the strains were selected from a collection of 154 strains from cows in dairy farms in Victoria and Queensland, Australia suffering from clinical and subclinical mastitis. The phenotypic characteristics that were used to select these 2 strains were capsule formation, biofilm forming potential, presence or absence of biofilm-associated genes including *ica*, *spa*, *bbp*, *hla*, and *hlb*. The reference *S. aureus* strains used in this study were strain M (CP1), Smith diffuse strain (CP2), USA 400 MW2 (CP8), Strain Newman and USA 100 NRS 648 (CP5), CP-negative isolates (USA 300 LAC and USA 300 NRS 648) and a strong biofilm-forming strain ATCC 29,213 as a positive control.

### 2.2. Capsular Typing of S. aureus 

Capsular typing of the *S. aureus* strains used in this investigation was carried out using molecular as well as serological methods as described elsewhere [9]. Briefly, Extraction of DNA from the 2 strains of *S. aureus* was accomplished using the extraction kit (MO BIO laboratories, Inc, Carlsbad, CA, USA). The PCR cycling parameters for *cap*1, *cap*5 and *cap*8 have been described previously [16]. For serotyping CP 1, 2, 5, and 8 specific antisera were produced in Quackenbush Swiss line 5 mice after gaining approval from the Animal Ethics Committee of Curtin University (Approval number: AEC_2011_65). The preparation of the vaccines and production of CP-specific sera were carried out according to the methods previously described [9]. A slide agglutination test was performed to determine the serotype of the 4 strains of *S. aureus*. Each strain was grown on Mueller Hinton (MH) agar plates at 37 °C overnight and a single colony was picked and suspended in a drop of 0.9% normal saline on a clean glass slide. A drop of serum was added to the suspension and checked for formation of agglutination within 20 s. The strains, which did not show agglutination against CP1, 2, 5 and 8—specific antisera were considered as non-typeable (Table 1). 

### 2.3. Determination of Biofilm Forming Potential of S. aureus 

Biofilm forming potential of the two NT *S. aureus* strains were determined by Congo red agar (CRA) method and Tissue Culture plate (TCP) method as described previously [16] (Table 1).

### 2.4. Detection of PIA-Dependent Biofilm Production Related Genes of S. aureus 

The *ica* typing of the *S. aureus* isolates were accomplished by using conventional PCR described elsewhere [37] (Table 1).

### 2.5. Detection of Virulence Genes of S. aureus 

Conventional PCR was carried out to detect biofilm related MSCRAMM and toxin genes of the two NT *S. aureus* strains. The primers, Tm, for all the MSCRAMM-encoding (*cna*, *clfA*, *clfB*, *spa*, *fnbpA, fnbpB*, *bbp*, *isdA*, *isdB*, *sdrD*, *sdrE* and *bap*) and toxin genes (*hla*, *hlb*, *eta*, *etb*, *pvl* and *tsst-1*) of the *S. aureus* strains used in this study have been described elsewhere [38] (Table 1).

### 2.6. Infection of Mammary Gland Using NT S. aureus Strains

#### 2.6.1. Animal Ethics Approval

All animal work described in this investigation was approved by the Animal Ethics Committee of Curtin University (Approval number: AEC_2012_14) prior to commencement of the experiment. The mice were used for the study ensuring compliance with the Western Australian Animal Welfare Act 2002.

#### 2.6.2. Preparation of Bacterial Inocula

The two NT *S. aureus* strains, *S. aureus* 83 and 87 were harvested on MH agar plates at 37 °C for 18 h. The colonies were washed from the plates using 20 mL of isotonic saline and suspended in isotonic saline to give a final viable bacterial count of 4 × 10^11^ mL^−1^ [39].

#### 2.6.3. Mice

A total of 12 Balb/c first-pregnancy mice, in three groups (such as strain 83, strain 87, control groups) comprising 4 mice in each group were used for the experiment. The 5–15 days old pups were removed from the lactating mice approximately 1 h prior to the experiment and euthanized.

#### 2.6.4. Method of Infection of the Mammary Gland

Infection of mammary glands using *S. aureus* 83 and 87 was carried out using a slightly modified procedure (Protocol S1) [40] described elsewhere [30]. Briefly, mice were anaesthetised using 100 mg kg^−1^ ketamine and 10 mg kg^−1^ xylazine administered by the intraperitoneal route and surrounding area of the fifth pair of mammary glands (L5 and R5) was disinfected with 70% ethanol. The duct orifice of the teat was located using a binocular dissecting microscope and 0.05 mL of bacterial suspension equivalent to 2 × 10^10^ CFU (Colony Forming Unit) *S. aureus* was injected using a blunt smooth 31-gauge hypodermic needle to a depth of not more than 4 mm. The mammary glands were harvested for 48 h and the mice were observed at six-hour intervals to assess development of macroscopic clinical signs of infection. The control group of mice was injected with normal saline following the same procedure.

#### 2.6.5. Post Inoculation Examination

##### Macroscopic Examination

The mice were monitored at an interval of 6 h for the clinical symptoms or any mortality. The 48 h post-infection was chosen for euthanasia as per the experimental mastitis model standerdised by Anderson and Chandler to study histological and bacteriological changes caused by *S. aureus* [39]. However, in our study none of the mice survived until 48 h. The level of clinical signs was graded as 0 (no macroscopic changes), + (low) grade, ++ (medium grade) and +++ (severe grade) based on the observed clinical features including redness, swelling, and discolouration of mammary gland, exudate, morbidity, and mortality (Table S1).

##### Bacteriological Procedure

Mammary Gland

After 48 h of infection, L5 mammary glands from both control and test mice were collected aseptically and processed for bacteriological load study [39]. The mammary glands were ground individually in sterile Griffith’s tubes containing 2 mL of sterile normal saline. The homogenates from the mammary glands were subjected to serial tenfold dilutions and inoculated on Baird Parker (BP) agar plates (Pathwest, Laboratory Medicine, WA) by the spread plate method and incubated at 37 °C for 48 h, followed by determination of colony counts of *S. aureus* per mammary gland. 

##### Blood, Liver, Lung, and Spleen

Blood samples obtained by cardiac puncture and organs including liver, lung and spleen homogenates were inoculated on BP agar plates and incubated at 37 °C for 48 h. 

##### Histological and Cytological Procedure

Mammary Gland

After 48 h of infection, R5 mammary glands were collected aseptically for histological examination [39]. Prior to embedding in paraffin wax, glands were fixed using 10% neutral buffered formalin for 24 h and processed on an automatic tissue processor. Sections were cut at 4 µm thickness at three levels and stained by the Haematoxylin and Eosin stain [41]. An additional section was stained for bacteria using the Gram Twort Method [42].

Blood

Blood smears were prepared following standard procedure and stained by the Diff Quik method [43].

##### Grading of Histological Changes Observed in Mammary Glands

The histopathological changes observed in mammary glands of mice, infected with *S. aureus* 83 and 87 were graded as follows:

**Level 0:** No reaction.

**Level 1:** Organisms identified with minimal inflammatory response in mammary tissue.

**Level 2:** Moderate inflammation in peri-mammary and intramammary tissue with intra luminal organisms observed.

**Level 3:** Marked inflammatory cell infiltration into mammary tissue in the presence of organisms with evidence of tissue degeneration including necrosis.

#### 2.6.6. Quantification of Inflammatory Cytokines 

BD cytometric Bead Array (CBA) Mouse/Rat soluble protein Master Buffer Kit (BD Biosciences), USA was used to quantify inflammatory cytokines, IL-1β, IL-6, IL-10, IL-12, IL-17A, IFN-γ and TNF-α in serum samples of mice. Standard protocol provided with the kit was used to prepare Mouse/Rat soluble protein flex set standards, capture beads and detection reagents. Briefly, 50 µL of Mouse/Rat soluble protein flex set standard dilutions ranging from 1:2 to 1:256 and one negative control containing only assay diluent was prepared. To 10 µL of each unknown serum sample, 10 µL of each capture bead and mixed PE (phycoerythrin) detection reagent was added. After adding capture beads and PE detection reagent tubes were incubated at 4 °C for 1 h each after in dark. Immediately after incubation, 200 µL of wash buffer was added to each tube and centrifuged at 200× *g* for 5 min. The supernatant was aspirated, discarded and the remaining pellet was reconstituted using 200 µL of wash buffer. This reconstituted pellet was used for acquiring on an Attune Acoustic Focusing Flow Cytometer (Thermofisher Scientific, Waltham, MA, USA). Samples were analysed using the FlowJo software.

#### 2.6.7. Statistical Analysis

Statistical analysis was carried out using Student’s *t*-test to compare total viable counts of *S. aureus* recovered from mammary glands injected with non-typeable strong biofilm forming *S. aureus* and weak biofilm forming *S. aureus*. The Student’s *t*-test was also performed to compare the IL-1β, IL-6, IL10, IL-12, IL-17, TNF-α and IFN-γ levels between groups of mice injected with *S. aureus* phenotypes. Statistical significance was set at *p* < 0.05. 

## 3. Results

### 3.1. Detection of Capsular Types of S. aureus

Both *S. aureus* 83 and 87 were found to be non-capsulated as these strains carried none of the three loci (*cap5, cap8* or *cap1*) and were not agglutinated by any of the CP-specific sera (CP 1, CP 2, CP 5 and CP 8) subjected to slide agglutination test. 

### 3.2. Determination of Biofilm Forming Potential of S. aureus Isolates 

#### 3.2.1. CRA and TCP Method

In both CRA and TCP methods, *S. aureus* strain 83 (OD value 0.775) was detected to be strong biofilm former in vitro whereas *S. aureus* strain 87 (OD value 0.367) was a weak biofilm former in vitro.

#### 3.2.2. *ica* Typing of *S. aureus* Isolates

*S. aureus* strain 83 confirmed PIA dependant biofilm formations by harbouring the *ica*A and *ica*D genes whereas these genes were not detectable in *S. aureus* strain 87. 

### 3.3. Detection of Different MSCRAMM-Encoding Genes of S. aureus Using Conventional PCR

Both *S. aureus* strains harboured genes encoding alpha and beta toxin. *S. aureus* 83 carried *clfA*, *clfB, spa, bbp, isdA, isdB, sdrD* and *sdrE* MSCRAMM genes. *S. aureus* 87 was found to carry *clfA, clfB, isdA, isdB, sdrD* and *sdrE* genes (Table 1).

### 3.4. Macroscopic Examination of Mammary Glands for Clinical Symptoms 

The control group of mice injected with normal saline did not show any clinical symptoms and the mammary glands appeared normal. Both the test groups of mice infected with *S. aureus* 83 and 87 strains showed medium grades of clinical symptoms. However, mice injected with *S. aureus* 83 died 24 h of post inoculation and the group injected with *S. aureus* 87 died 30 h post inoculation (Table 2).

### 3.5. Bacterial Load and Histopathological Changes of Mammary Gland

The log average number of bacteria (CFU) isolated from the mammary glands of all the 3 groups of mice including control group and the associated histopathological changes in the mammary glands are presented in Table 3. The bacterial load of mammary glands of mice infected with *S. aureus* 83 was significantly higher (*p* < 0.05) with 8.23 ± 0.001 CFU compared to those infected with *S. aureus* 87 which was 7.91 ± 0.003 CFU.

#### 3.5.1. Bacteriology of Blood and Histopathology of Liver, Lung, and Spleen

The culture of blood and organs (liver, lung and spleen) in BP agar plates was negative for *S. aureus* indicating no evidence of systemic infection. There was no evidence of inflammation in tissue sections of lung, liver and spleen from any of the mice.

#### 3.5.2. Histopathology of Mammary Glands Post-infection with Biofilm Forming *S. aureus*

No evidence of inflammatory response was recorded in mammary tissue of control mice which were inoculated with sterile normal saline. The mammary tissue of all the mice infected with *S. aureus* 83 and 87 demonstrated identical Level 1 inflammation.

### 3.6. Quantification of Inflammatory Cytokines in Serum

Quantification study of inflammatory cytokines, IL-1β, IL-6, TNF-α and other cytokines including IL-10, IL-12, IL-17A and IFN-γ showed that the levels of IL-1β, IL-6, IL-12, IL-17 and IFN-γ were significantly higher (*p* < 0.05) in the sera of mice inoculated with non-typeable strong biofilm forming *S. aureus* 83 than those inoculated with weak biofilm forming non typeable *S. aureus* 87 (Table 4).

## 4. Discussion

Globally, *S. aureus* remains one of the predominant causes of clinical and subclinical mastitis in dairy ruminants. Non-typeable strains of *S. aureus* can survive in the mammary gland for longer duration than the encapsulated strains [2,8]. Higher degree of inflammation appeared to have been induced by encapsulated *S. aureus* than the non-typeable *S. aureus* strains, which leads to quick clearance of these cells by the host immune system. The non-typeable cells are quickly internalized by the mammary epithelial cells due to absence of capsule and thus they are protected from the action of phagocytic cells [8] and allows bacteria to persist leading to chronic infection [44]. Biofilm producing *S. aureus* can attach more effectively to the epithelial lining of mammary glands to develop intra-mammary infection [31]. In fact, *S. aureus* isolated from mammary glands are more likely to form biofilm than *S. aureus* isolated from external sources including milking machines [45]. Bacteria growing in biofilm demonstrate increased resistance to antimicrobial therapy [14] due to delayed penetration of antimicrobial agents crossing the barrier of slimy biofilm matrix, modification in the growth rate of pathogens residing in biofilm and certain physiological and genotypic changes in pathogen residing in biofilm [46]. Furthermore, the ability of the macrophages to invade into biofilm is limited and the pathogen in biofilm is able to polarise the macrophages from proinflammatory microbicidal M1 phenotype to an M2 phenotype which exert anti-inflammatory properties and restrict phagocytosis [28,47]. To make the situation worse, in a biofilm environment, *S. aureus* initiate a favourable interaction with the Myeloid derived suppressor cells (MDSCs) which exert immunosuppressive properties [48] and this phenomenon is partly aggravated by cytokines such as IL10 [49] and IL12 [50]. As the NT *S. aureus* can remain longer in the mammary epithelial cells well protected from host phagocytosis and additionally the biofilm formation is one of the important survival strategies of the pathogen [51] in intramammary infection, it is important to understand the magnitude of mammary tissue damage post infection by NT *S. aureus* with different strength of biofilm forming potential for future therapeutic interventions. 

In the current study, both the NT strains of *S. aureus* with different strength of biofilm formation produced identical clinical symptoms and mammary tissue damage. Both the strains produced moderate level of clinical symptoms of mastitis and level 1 histopathological lesion in the mammary gland. However, the bacterial load in the mammary glands of mice injected with the non-typeable *S. aureus* 83 strain with strong biofilm forming ability was significantly higher (*p* ≤ 0.05) than the weak biofilm forming *S. aureus* 87. The period of observation in the study was 48 h. Both the test groups of mice died before 48 h. The mice injected with *S. aureus* 83 (strong biofilm former) survived only 24 h followed by the mice injected with *S. aureus* 87 (weak biofilm former). Mice in the latter died at 30 h post inoculation. The analysis of sera samples collected from mice immediately before death showed higher levels of IL-1β, IL-6, IL-10, IL-17A, IFN-γ and TNF-α (Table 4). In one study, it was suggested that quick internalization of non-typeable *S. aureus* cells by the mammary epithelial cells may have prevented the clearance of *S. aureus* from the mammary gland providing scope for production of high levels of cytokines of different types [52]. The highly elevated level of various cytokines can lead to cytokine storm, a fatal immune response which may result in sudden death [53,54]. It has been demonstrated that patients who died due to cytokine storm had higher levels of anti-inflammatory cytokine, IL-10 and pro inflammatory cytokines, IL-1β, IL-6 and TNF-α in serum samples [55]. In the present study, between both the groups of test mice, the mice injected with *S. aureus* 83 produced significantly higher levels of IL-1β, IL-6, IL-12, IL-17 and IFN-γ (*p* < 0.05) than the mice injected with *S. aureus* 87. The level of IL-6 in sera samples of mice injected with *S. aureus* 83 was extremely high (15479.9 ± 532 Pg/mL) which could have been responsible for the death of the mice within mere 24 h post inoculation. In addition, IL-12 is a cytokine with both pro and anti-inflammatory effects, can assist in the recruitment of MSDCs. IL-10, an anti-inflammatory cytokine mainly produced by the MSDCs during *S. aureus* biofilm infection is capable of promoting the growth of biofilm [49]. In the current study, IL-10, IL-12 and MSDCs may be the contributing factors for the development of anti-inflammatory environment in the mammary gland resulting only Grade 1 mammary tissue damage. However, this warrants further investigation.

The role of biofilm in complication of bovine mastitis has been established previously [30,46,56]. In this study, the only phenotypic difference between both the strains was the biofilm forming ability. Though not conclusively, it can be suggested that biofilm forming ability of non-typeable *S. aureus* may play role in the virulence of *S. aureus* as increased higher levels of PIA/PNAG-associated biofilm could be helpful in dispersion of biofilm facilitated by bacterial cell to cell interaction in the mammary gland [57] leading to higher colonisation of the pathogen in mammary tissue [31] and possibly systemic dissemination of infection [56]. This might have resulted in higher bacterial load and significantly higher levels of cytokines in mice infected with strong biofilm producing *S. aureus* which might have contributed to the mortality of the mice. In the mammary glands, inflammation has been associated with neutrophil chemo-attractants and the cytokines (IL-1β, IL-6, TNF-α and IL8) [58,59]. Local induction of cytokines including IL-1β and IL- 6 post infection of the mouse mammary gland with *S*. *aureus* was reported by Breyne and co-workers [32]. IL-6 in quarter milk has been proven as a prediction marker of bovine mastitis [60]. In the current study, the extraordinarily high amount of IL-6 in sera samples of mice infected with biofilm forming NT *S. aureus* suggests an important role of IL-6 in virulence of strong biofilm forming *S. aureus* in mouse mastitis model. It will be interesting to investigate the neutralisation effect of IL-6 by anti-IL-6R antibody in preventing mortality in mice in the future investigations. 

Due to non-availability of commercial mutant NT *S. aureus* strains of different biofilm forming potential, we have emphasized on using clinical isolates and included selected biofilm related phenotypic characteristics to be compared in the selected two *S. aureus* strains used in this study. This is one of the limitations of our study. Further investigation will be important to compare the virulence and pathogenicity of mutant *S. aureus* strains that are genotypically and phenotypically similar but with different biofilm forming abilities. Additionally, the expression of *S. aureus* biofilm in vitro may not correlate with the expression that occurs in vivo in the mammary gland. The future research will focus on comparing the in vitro attachment potential of NT *S. aureus* strains (used in this study) to bovine mammary epithelial cells (MAC-T) to in vivo response in either a mouse mastitis model or in dairy cows. It will be interesting to replicate this study using *S. aureus* with different biofilm forming potential from different species to understand if there are any species-specific differences to the host response. 

The potential information generated from this study will contribute new knowledge in mastitis pathology associated with NT *S. aureus* and may aid in future research to strategize different treatment options with advanced pharmacological interventions to reduce tissue damage aiming to control mastitis.

## 5. Conclusions

From this study it was concluded that phenotypic PIA-dependent biofilm production by clinical non-typeable *S. aureus* is not associated with the intensity of inflammation in mammary gland. Clinical non-typeable *S. aureus* strains isolated from bovine mastitis cases developed identical moderate clinical symptoms, Grade-1 mammary tissue damage and generated minimal inflammatory infiltrates in the mammary tissue when tested in a mouse mastitis model.

## Figures and Tables

**Table 1:** Phenotypic characteristics of *Staphylococcus aureus* strains used in the study.

Sl No	*S. aureus* Strain	Capsular Polysaccharide Type	Biofilm Formation (TCP */CRA ** Method)	Presence of *ica* Genes *icaA* *icaD*	*spa* Gene	*bbp* Gene	Presence of Alpha (*hla*) and Beta (*hlb*) Toxin Genes
1	*S. aureus* 83	Non-capsulated	Strong biofilm (OD *** 0.775)	+ve +ve	+ve	+ve	hla, hlb
2	*S. aureus* 87	Non-capsulated	Weak biofilm (OD 0.367)	−ve −ve	−ve	−ve	hla, hlb

* TCP: tissue culture plate; ** CRA: Congo red agar; *** OD: optical density.

**Table 2:** Clinical signs observed in different groups of mice post-infection (observations up to 30 h post inoculation).

Time Post Inoculation	*S. aureus* 83				*S. aureus* 87				Normal Saline (Control)			
	P*1	P2	P3	P4	P1	P2	P3	P4	P1	P2	P3	P4
**6 h**	0	0	0	0	0	0	0	0	0	0	0	0
**12 h**	+	+	+	+	+	+	+	+	0	0	0	0
**18 h**	++	++	++	++	+	+	+	+	0	0	0	0
**24 h**	++/D	++/D	++/D	++/D	++	++	++	+	0	0	0	0
**30 h**	-	-	-	-	++/D	++/D	++/D	++/D	0	0	0	0

* P: pair; fifth pair of mammary glands (L5 and R5) in each mouse; clinical features include redness, swelling, and discolouration of mammary gland, exudate, morbidity, and mortality; grade scores compare observed features to the most severe changes: 0 no macroscopic changes, + low grade, ++ medium grade, +++ severe grade.

**Table 3:** Total viable counts of NT *S. aureus* recovered from mammary glands after death of mice and histopathology of mammary glands.

Group	Total Number of Mammary Glands ^1^ Investigated	Log Average Number of Bacteria (CFU ^3^) Recovered from Mammary Glands ± SE	Histopathology Grade			
			M ^4^1	M2	M3	M4
*S. aureus* 83	4	8.23 ± 0.001 *	1	1	1	1
*S. aureus* 87	4	7.91 ± 0.003	1	1	1	1
Control (NS ^2^)	4	0	0	0	0	0

* *p* < 0.05, the comparison of results of bacterial load was undertaken between *S. aureus* 83 and *S. aureus* 87 groups; ^1^ L5 and R5 mammary glands were used for bacteriological and histological procedures, respectively; ^2^ NS = normal saline; ^3^ CFU = colony forming unit: ^4^ M = mammary gland.

**Table 4:** Detection of levels of different cytokine biomarkers in sera samples of mice before death.

Group	*S. aureus* Phenotype	IL-1β Pg/mL ± SE	IL-6 Pg/mL ± SE	IL-10 Pg/mL ± SE	IL-12 Pg/mL ± SE	IL-17A Pg/mL ± SE	IFN-γ Pg/mL ± SE	TNF-α Pg/mL ± SE
1	*S. aureus* 83	321.7 * ± 23	15479.9 * ± 532	66.8 * ± 0.96	3.0 * ± 0.42	28.6 * ± 1.79	59.5 * ± 1.78	163.3 ± 4.5
2	*S. aureus* 87	27.7 ± 41	529 ± 109	12.86 ± 0.69	1.43 ± 0.35	18.20 ± 0.5	12.70 ± 1.50	174.9 ± 21
3	Control (NS)	0	0	0	0	0	0	0

* *p* < 0.05, the comparison of results of inflammatory cytokines was undertaken between *S. aureus* 83 and *S. aureus* 87 groups.

## Data Availability

All the data generated from this research are available upon reasonable request.

## Supplementary Materials

The following Supplementary Materials are being published alongside the article. The following are available online at https://www.mdpi.com/article/10.3390/ani11113047/s1, Protocol S1: Method of infection the mammary gland; Table S1: Grading of clinical signs.
