# Supplementary material for: Phenotypic PIA-Dependent Biofilm Production by Clinical Non-Typeable Staphylococcus aureus Is Not Associated with the Intensity of Inflammation in Mammary Gland: A Pilot Study Using Mouse Mastitis Model
Source: Animals (Basel). 2021 Oct 25;11(11):3047. doi: 10.3390/ani11113047 (PMC8614318; doi:10.3390/ani11113047)
Supplement: Supplementary file 1 [file animals-11-03047-s001.zip › animals-1375606-supplementary.pdf]

Protocol S1 <u>Method of infection of the mammary gland</u>

Inoculation of the different *S. aureus* phenotypes into mammary glands was carried out using a modification to the procedure described previously by Brouillette et al., 2004. In our modified method, we eliminated the initial step of incision of the proximal end of the teat to expose the teat canal. Instead of incising the proximal end of the teats, we inoculated the *S. aureus* strains directly into the mammary duct. The details of the method are provided below.

(i) Balb/c first-pregnancy mice were used for this study.
(ii) Pups were removed from the lactating mice when they were between 5-15 days old, approximately 1h prior to the experiment.
(iii) They were euthanised immediately after removing from their mothers.
(iv) Mice were anaesthetised using 100 mg kg-1 ketamine and 10 mg kg-1 xylazine administered by the intraperitoneal route and laid in the supine position.
(v) All animals were administered one dose of Buprenorphine hydrochloride (0.05-0.1 mg kg^{-1}) subcutaneously pre-operation so that anticipated pain relief was available for up to 12 h post bacterial inoculation.
(vi) The area surrounding the 5th pair of mammary glands (L5 and R5) was disinfected with 70% ethanol.
(vii) A binocular dissecting microscope was used to locate the duct orifice of the teat.
(viii) While controlling the teats with sterile forceps, 0.05 ml of bacterial suspension equivalent to o 2x 10^{10} CFU *S. aureus* was injected using a blunt smooth 31-gauge hypodermic needle to a depth of not more than 4mm.
(ix) The control group of mice was injected with normal saline using the same procedure.
(x) The infection was allowed to progress for 48 h.
(xi) Mice were observed at six-hour intervals to assess development of macroscopic clinical signs of infection

Table S1. <u>**Grading of clinical signs**</u>

Clinical signs grades	Redness	Swelling	Discoloration of mammary gland	Exudate	Morbidity	Mortality
0	-	-	-	-	-	-
+	+	-	-	-	-	-
++	+	+	-	-	+	-
+++	+	+	+	+	+	±

Assignment of zero (0) denotes no inflammation, + denoted low grade, ++ denoted medium grade and +++ severe inflammation using redness, swelling, discoloration of mammary gland, presence of exudate in the teat, morbidity, and mortality.
